# Clinical anatomy of the paranasal sinuses and its terminology

**DOI:** 10.1007/s12565-023-00745-3

**Published:** 2023-10-09

**Authors:** Piotr Paweł Chmielewski

**Affiliations:** Division of Anatomy, Department of Human Morphology and Embryology, Faculty of Medicine, Wroclaw Medical University, Wrocław, Poland

**Keywords:** Paranasal sinuses, Anatomical terminology, Anatomical nomenclature, Human gross anatomy, Clinical anatomy

## Abstract

Since its inception, the International Anatomical Terminology has been an indispensable and widely embraced resource for authors, anatomists, researchers, and medical professionals, ensuring standardized anatomical terminology across various disciplines. Nonetheless, it is widely acknowledged that periodic updates and enhancements are necessary to incorporate the latest scientific knowledge and advancements in imaging techniques. The current version of Terminologia Anatomica includes a section dedicated to the paranasal sinuses, encompassing ethmoidal cells and three sinuses: frontal, sphenoidal, and maxillary. However, the anatomical lexicon pertaining to the paranasal sinuses is more extensive. In clinical practice, multiple terms related to clinically significant structures are commonly employed. This article focuses on the clinical terminology associated with the paranasal sinuses, proposing significant extensions to the existing Terminologia Anatomica. These extensions aim to enrich the anatomical nomenclature and facilitate a harmonious convergence between the language of clinicians and the anatomical lexicon. Further endeavors should bridge the gap in anatomical nomenclature and improve communication between anatomists, researchers, and clinicians, thereby enhancing diagnostic accuracy and improving interdisciplinary research collaboration.

## Introduction

Since its first edition, the Terminologia Anatomica (Federative Committee on Anatomical Terminology 1998) has been an invaluable and widely embraced resource for anatomists, authors, researchers, and medical professionals, ensuring consistent and standardized anatomical terminology across diverse scientific disciplines (Whitmore 1999; Kachlik et al. 2008, 2015; Strzelec et al. 2017). In 2020, however, the General Assembly of the International Federation of Anatomical Associations (IFAA) adopted and promulgated the second edition of the Terminologia Anatomica (Federative International Programme for Anatomical Terminology 2019). Notwithstanding this adoption, most European anatomical associations and their leading publications continue to adhere to the former edition (TA 1998) rather than the latter.

Nevertheless, many authors argue that certain sections of both TA 1998 and TA 2019 lack the necessary level of detail and precision (Kachlik et al. 2008, 2010, 2016, 2017, 2018, 2021; Musil et al. 2019). Most notably, the sections dedicated to the paranasal sinuses and vasculature are considered insufficiently comprehensive (Chmielewski 2023). Indeed, despite their significant clinical importance, these structures have not received adequate attention in terms of appropriate and meticulous naming. Therefore, further updates and enhancements to the anatomical nomenclature are deemed necessary to meet the terminological requirements of clinical practice (Chmielewski 2020; Neumann et al. 2020; Chmielewski and Domagała 2020; Chmielewski and Strzelec 2020).

The anatomy of the paranasal sinuses, intricately intertwined with adjacent structures, holds paramount importance in radiology, clinical medicine, and surgical interventions, particularly in functional endoscopic sinus surgery (Stammberger and Kennedy 1995; Arslan et al. 1999; Gupta et al. 2012). It is often argued that precise knowledge of the anatomy of the paranasal sinuses is essential in ensuring safe and successful outcomes during such procedures (Vaid and Vaid 2022). The prevalence of pathological conditions, such as sinusitis, nasal polyps, and head tumors, emphasizes the significance of understanding the intricacies of their anatomy and pathology. In fact, the paranasal sinuses exhibit a wide range of variations (Kantarci et al. 2004; Cellina et al. 2020), and “dangerous” variants, such as the presence of Onodi’s cells, Haller’s cells, as well as a low placed fovea ethmoidalis are of paramount importance (Gupta et al. 2012), as anticipating and identifying these variants before surgery are crucial to avoid the risk of injury. A comprehensive understanding of the paranasal sinuses is indispensable for accurate diagnosis and effective disease management, making it a vital prerequisite for both precise diagnosis and the development of appropriate treatment strategies.

This article concentrates on the clinical terminology associated with paranasal sinuses, proposing substantial extensions to the existing Terminologia Anatomica. These proposed extensions have the potential to enrich the anatomical nomenclature. In fact, the integration of these proposed extensions into forthcoming editions of the Terminologia Anatomica aims to foster a harmonious convergence between the anatomical lexicon and the domain of clinical medicine, thereby enhancing interdisciplinary communication and knowledge transfer.

## Frontal sinus drainage pathway

The “frontal sinus drainage pathway” (*ductus sinus frontalis, ductus frontonasalis*, or simply *ductus frontalis*; see Table 1) is composed of two distinct segments: the frontal ostium superiorly and the frontal recess inferiorly (Dassi et al. 2020). The frontal ostium is bounded by the anterior ethmoidal cells anteriorly, the roof of the ethmoidal bulla posteriorly, the orbital plate (“lamina papyracea”) laterally, and the basal lamella of the middle nasal concha medially. The frontal recess (*recessus frontalis*) is a space situated within the nasal cavity, often described as an inverted cone-shaped or funnel-like recess representing the superior portion of the ethmoidal infundibulum (Musil et al. 2019). This recess is commonly found in the vast majority of individuals (not a variant, but a typical structure) and serves as a connection between the frontal sinus and the middle nasal meatus. The initial characterization of this space is attributed to Killian, with subsequent valuable insights contributed by van Alyea regarding its pneumatization.

**Table 1:** Anatomical terminology pertaining to the paranasal sinuses that can be incorporated into future editions of Terminologia Anatomica

Latin term	English equivalent	Frequency of variant
Cellula sphenoethmoidalis	Sphenoethmoidal cell	4–65%
Cellula interlamellaris	Interlamellar cell	
Cellula infraorbitalis	Infraorbital ethmoidal cell	4–20%
Columella ethmoidalis	Ethmoidal column (“vertical bar”)	
Complexus ostiomeaticus	Ostiomeatal complex	
Concha bullosa	Concha bullosa of middle nasal concha	17–36% (50% in Turkish)
Ductus frontalis	Frontal duct (“frontal sinus drainage pathway”)	
Fovea ethmoidalis ossis frontalis	Fovea ethmoidalis of frontal bone	
Lamellae basilares	Basal (or basilar) lamellae	
Recessus frontalis	Frontal recess	
Recessus opticocaroticus lateralis	Lateral opticocarotid recess	
Recessus opticocaroticus medialis	Medial opticocarotid recess	
Recessus retrobullosus	Retrobullar recess	94%
Recessus suprabullosus	Suprabullar recess	71%
Recessus terminalis	Terminal recess (of ethmoidal infundibulum)	49–85%
Sinus lateralis	Lateral sinus	> 70%

**Fig. 1:**
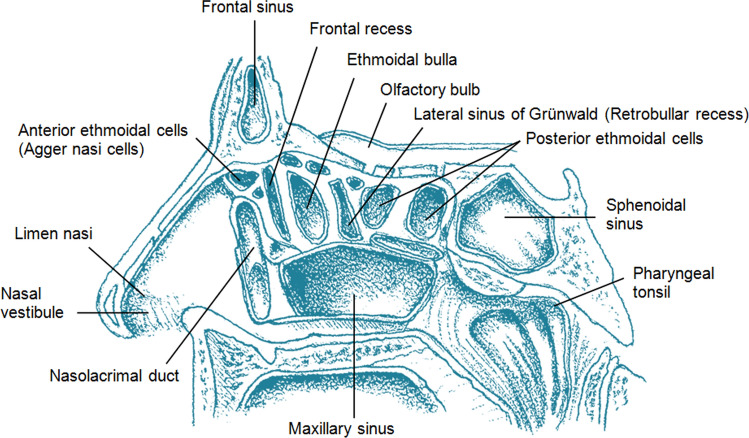
A sagittal section through the lateral wall of the right nasal cavity, highlighting the frontal recess and the lateral sinus

Anteriorly, the frontal recess is demarcated by the anterior ethmoidal cells (“agger nasi cells”), which constitute the most anterior cells within the ethmoidal air cells. Medially, its boundaries are established by the middle nasal concha, while its lateral border is formed by the orbital plate (formerly referred to as the “lamina papyracea”). Posteriorly, this recess is defined by the ethmoidal bulla. The presence of the posterior wall of this recess depends on whether the basal lamella of the ethmoidal bulla reaches the cranial base, separating the frontal recess from the retrobullar recess, which is a part of the lateral sinus (Fig. 1).

**Fig. 2:**
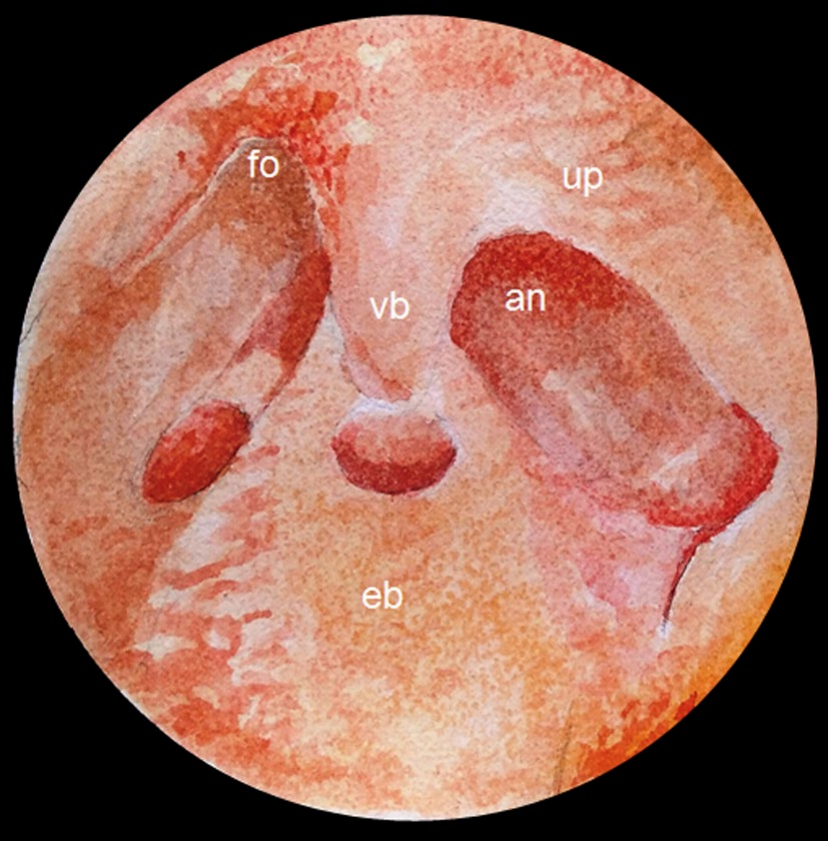
Endoscopic anatomy of the left frontal recess. The frontal ostium (fo) is the upper part of the frontal sinus drainage pathway. The vertical bar (vb) represents the union of the anterior ethmoidal cell with the uncinate process (up). The agger nasi (an) is situated anteriorly, and the ethmoidal bulla (eb) marks the posterior boundary of this recess

Due to its intricate architecture, the frontal recess ranks among the most complex anatomical structures found within the paranasal sinuses (Fig. 2), warranting recognition in future editions of the international standardized anatomical nomenclature. As Peng et al.’s (2022) stress, the frontal recess cells have an extremely intricate anatomical structure and can be divided into agger nasi cells, suprabullar cells, supra agger cells, supra agger frontal cells, supraorbital ethmoidal cells, frontal septal cells, and suprabullar frontal cells (Bent et al. 1994; Kuhn 1996).

In cases where the frontal recess is extensively pneumatized or when the attachment of the basal lamella protrudes, this recess takes on a tubular shape. This is why it used to be described as the “nasofrontal” duct or canal (*ductus nasofrontalis*, *canalis nasofrontalis*). However, these names are misleading and erroneous from a terminological standpoint and, therefore, they should be avoided (Musil et al. 2019).

## Terminal recess

The terminal recess (*recessus terminalis*) is a variable space within the ethmoidal infundibulum that protrudes ventrally from the point of the opening of the frontal sinus (Musil et al. 2019). In the case of an anomalous insertion of a vertical bony lamella, which is referred to as the uncinate process of the ethmoidal bone (*processus uncinatis ossis ethmoidalis*), the frontal recess is distinct and separate from the ethmoidal infundibulum.

## Agger nasi cells

The existing versions of the Terminologia Anatomica (Federative Committee on Anatomical Terminology 1998; Federative International Programme for Anatomical Terminology 2019) include the term anterior ethmoidal cells (Fig. 1). In clinical jargon, however, the “agger nasi cells” are the most anterior ethmoidal air cells that demarcate the anterior limits of the frontal recess and are often used as a surgical landmark for accessing this recess. As Gupta et al. (2012) point out, the agger nasi cells may narrow the frontal recess and obstruct the inferior part of the frontal sinus drainage pathway, leading to disease or complications.

## Vertical bar

Interestingly, the union of the anterior ethmoidal cell with the uncinate process, which is often referred to as the “vertical bar” (Fig. 2), is a very useful landmark to achieve a correct identification of the frontal recess and frontal sinus drainage pathway during endoscopic procedures (Dassi et al. 2020). This structure could be named *columella ethmoidalis* (ethmoidal column; Table 1), as the name “vertical bar” is not particularly appropriate or informative.

## Lateral sinus

**Fig. 3:**
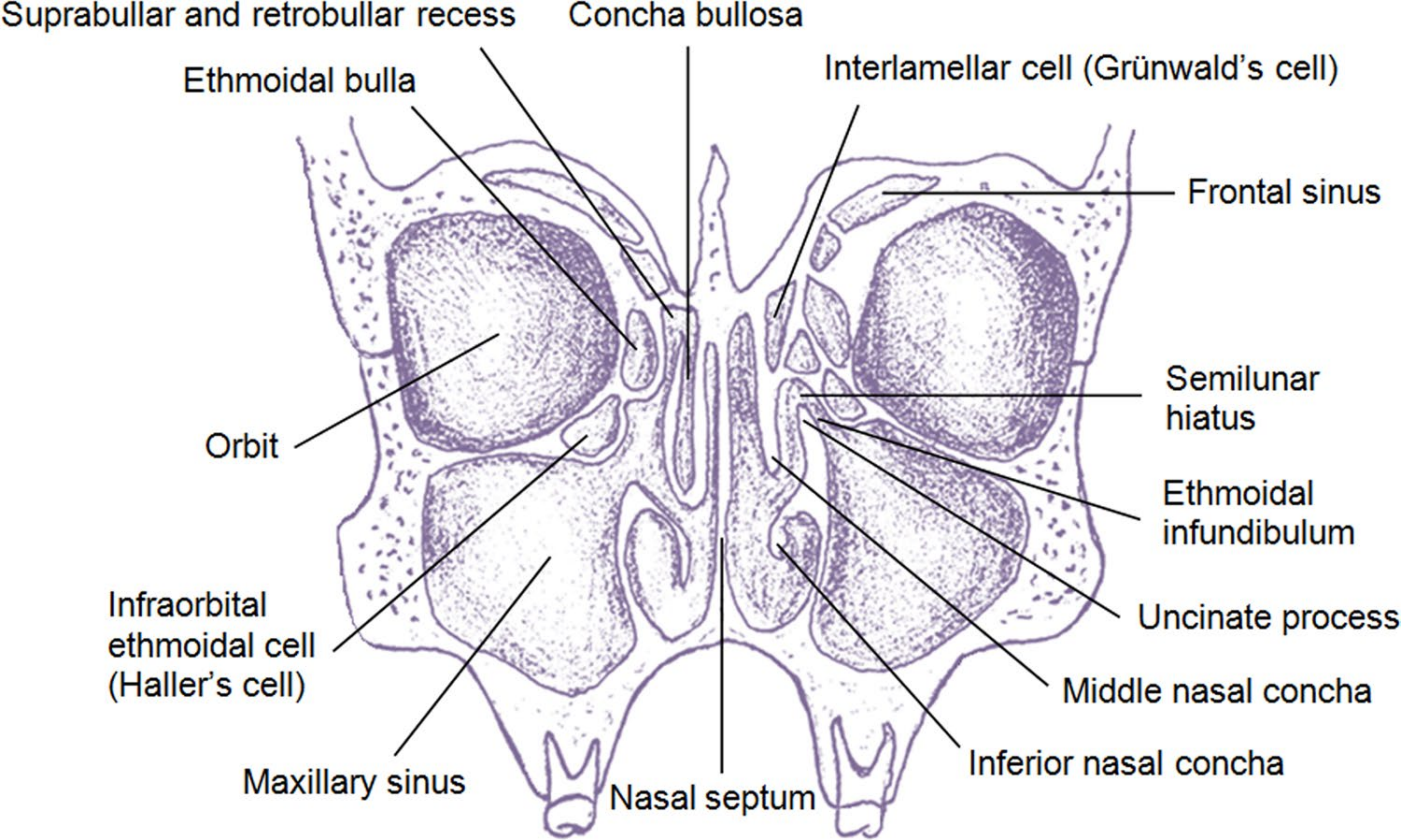
Topography and variants of the ethmoidal cells. Frontal section through the cranium at the level of the uncinate process. On one side of the nasal septum is the concha bullosa, whereas on the other side is the pneumatized vertical lamella of the middle nasal concha (the interlamellar cell). The infraorbital ethmoidal cells and the lateral sinus are also visible

The lateral sinus of Grünwald, also known as the lateral recess of the ethmoidal sinus, is a pneumatized space located within the nasal cavity, positioned behind and superior to the ethmoidal bulla (Stammberger and Kennedy 1995). It is bounded by several structures (see Figs. 1, 3): the roof of the ethmoid superiorly, the ethmoidal bulla (also known as the “ethmoid bulla” or the “promontory”, which represents pneumatization of the second basal lamella) anteriorly, the basal lamella of the middle nasal concha posteriorly, the middle nasal concha (“middle turbinate”) medially, and the orbital plate laterally.

The lateral sinus can be further subdivided into two distinct recesses (*recessus retrobullaris s. retrobullosus et recessus suprabullaris s. suprabullosus*), namely the retrobullar recess and the suprabullar recess (Musil et al. 2019). The former recess (nearly constant, 94%) is bounded by the ethmoidal bulla, the lateral aspect of the middle nasal concha, and the orbital plate, while the latter is situated between the ethmoidal bulla and the fovea ethmoidalis. It is noteworthy that the superior part of the suprabullar recess often contains the anterior ethmoidal artery.

Understanding the anatomy of the lateral sinus holds great clinical significance, particularly in the context of surgical interventions. Knowledge of its boundaries, recesses, and relationships with adjacent structures is essential for ensuring safe and precise navigation during clinical procedures involving the lateral wall of the nasal cavity. Surgeons oftentimes rely on this information to achieve optimal outcomes in managing conditions affecting the paranasal sinuses. Access to both portions of the lateral sinus, namely the suprabullar recess and the retrobullar recess, can be achieved through the medial and interior routes via the semilunar hiatus (Fig. 3). This anatomical comprehension is crucial for enhancing surgical efficacy and reducing the risk of complications, making it an indispensable aspect of clinical practice regarding paranasal sinus surgery.

## Concha bullosa

The term “concha bullosa” refers to the pneumatization of the middle nasal concha, occasionally extending to the superior nasal concha, with typical origins from the frontal recess of agger nasi (Stallman et al. 2004). It is crucial to differentiate this structure from the interlamellar cell of Grünwald (Fig. 3), which arises from the pneumatization of the vertical lamina of the middle nasal concha, extending from the middle nasal meatus. Recent anatomical studies have suggested a potential link between concha bullosa and nasal airway obstruction, implying that the presence and extend of pneumatization might significantly impact airflow dynamics, thereby contributing to respiratory symptoms (Tiwari and Goyal 2019). Gawlikowska-Sroka et al. (2016) argued that concha bullosa, which is a common anatomic variant (El-Din et al. 2021), may predispose to the paranasal sinusitis. However, Calvo-Henríquez et al. (2019), who presented a novel classification according to the axial extension of pneumatization, did not find a link between concha bullosa and chronic rhinosinusitis. Further investigations into the functional implications of concha bullosa hold the promise of providing valuable insights into the pathophysiology of nasal congestion, ultimately aiding in the development of more effective treatment strategies.

## Infraorbital ethmoidal cell

The infraorbital ethmoidal cell, also known as Haller’s cell, represents the pneumatization of the superior aspect of the maxillary sinus and the inferior wall of the orbit (Stammberger and Kennedy 1995). This cell occupies the space below the ethmoidal bulla and extends along the inferior part of the orbital plate (Fig. 3). The presence of this cell also poses a potential risk for iatrogenic injury if not accurately identified and accounted for during preoperative assessments. Surgeons must exercise caution and employ anatomical knowledge to ensure safe and successful interventions in this area. A comprehensive understanding of the anatomy and variations of the infraorbital ethmoidal cell is critical for minimizing the risk of unintended complications during surgical procedures.

## Sphenoethmoidal cell

**Fig. 4:**
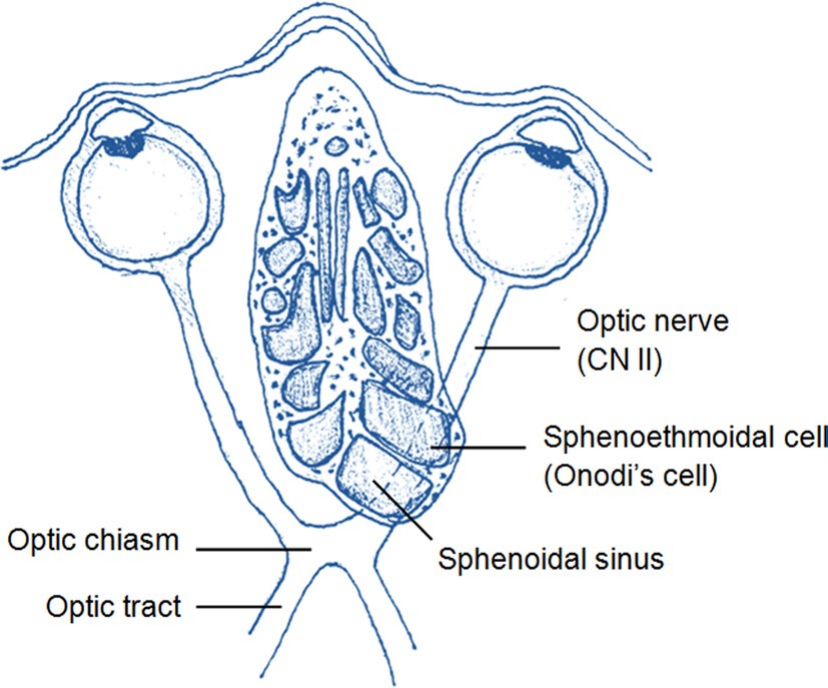
Topography and variants of the ethmoidal cells. Horizontal section through the ethmoidal bone at the level of the optic canal, showing the Onodi–Grunwald’s cell. If present, this cell protrudes backwards over the sphenoidal sinus, closely neighboring the optic nerve and internal carotid artery

The sphenoethmoidal cell, commonly known as Onodi’s cell (Ónodi-Grünwald cell), represents a posterior ethmoidal air cell that pneumatized in a posterolateral direction relative to the sphenoidal sinus (Fig. 4). Importantly, it is noteworthy that the optic nerve (CN II) and the internal carotid artery (ICA) are in close anatomical proximity to the sphenoethmoidal cell (Stammberger and Kennedy 1995). Thus, failure to anticipate the presence of this cell during surgical procedures can lead to disorientation and can increase the risk of optic nerve and ICA injury. Therefore, caution must be exercised when accessing the sphenoidal sinus through the ethmoidal cells, particularly during endoscopic sinus surgery, to minimize the potential risk to the optic nerve.

## Ostiomeatal complex

**Fig. 5:**
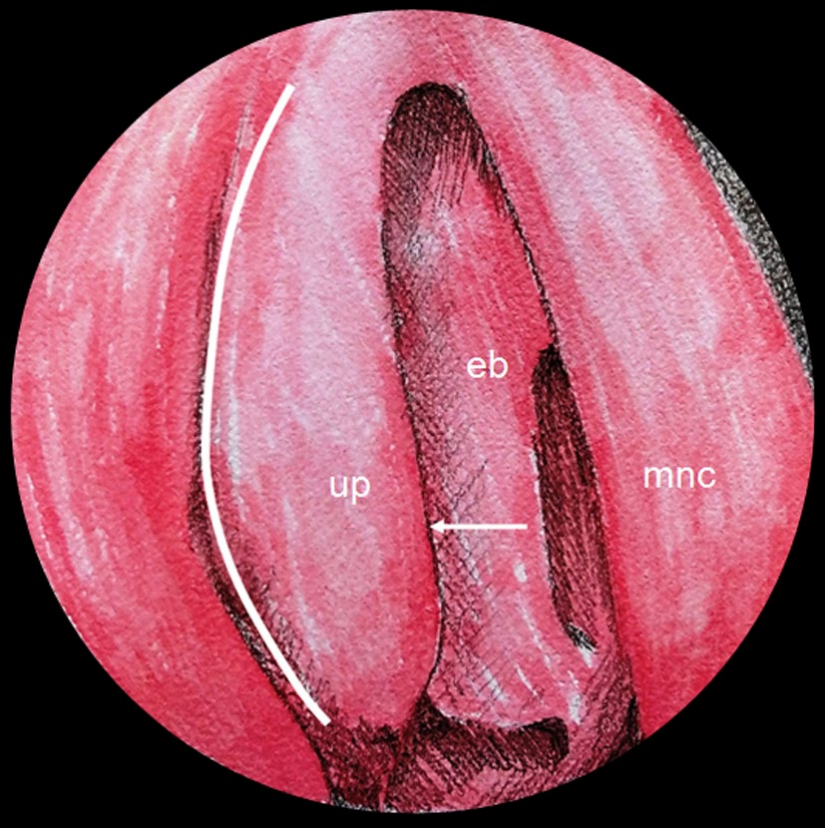
Endoscopic anatomy of the right ostiomeatal complex, highlighting the uncinate process (up), ethmoidal bulla (eb), and middle nasal concha (mnc). The semilunar hiatus (white arrow) and the approximate limit of the uncinectomy (white line) may also be identified

The ostiomeatal complex (albeit some authors use the name “osteomeatal complex”, even though this is a misnomer, cf. Waschke et al. 2019) is a functional unit within the nasal cavity, facilitating drainage from the frontal sinus, maxillary sinus, and ethmoidal sinus (Fig. 5). A comprehensive understanding of its anatomy is essential for mastering the fundamental principles of endoscopic surgical techniques. The ostiomeatal complex is demarcated by the middle nasal concha medially, the orbital plate laterally, and the basal lamina of the middle nasal concha posteriorly. This complex includes several critical structures, including the agger nasi, frontal recess, ethmoidal infundibulum, ethmoidal bulla, and anterior ethmoidal cells.

For clinicians engaged in endoscopic sinus surgery, a thorough knowledge of the ostiomeatal complex is indispensable, as it serves as a pivotal point of access and a crucial area for targeted interventions. Successful surgical outcomes, optimal disease clearance, and restoration of normal sinus function are contingent upon a profound understanding of the intricate interplay among its components. Therefore, recognizing the anatomical nuances and variations of the ostiomeatal complex significantly contributes to refining surgical techniques and elevating the quality of patient care. Emphasizing the importance of comprehending this complex structure can profoundly enhance the effectiveness of endoscopic sinus surgery.

## Conclusions

To conclude, the current version of anatomical nomenclature pertaining to the paranasal sinuses requires updates and enhancements to cover all important terms commonly used by medical professionals in clinical practice, ensuring precise communication and accurate documentation in medical settings. The current version of Terminologia Anatomica lacks multiple essential anatomical terms related to the paranasal sinuses, hindering comprehensive and standardized communication among researchers and medical experts. This article has addressed some of these lacking, proposing essential additions to Terminologia Anatomica. Several candidate names have also been outlined.
